# Occupational Exposures to Ebola Virus in Ebola Treatment Center, Conakry, Guinea

**DOI:** 10.3201/eid2308.161804

**Published:** 2017-08

**Authors:** Hélène Savini, Frédéric Janvier, Ludovic Karkowski, Magali Billhot, Marc Aletti, Julien Bordes, Fassou Koulibaly, Pierre-Yves Cordier, Jean-Marie Cournac, Nancy Maugey, Nicolas Gagnon, Jean Cotte, Audrey Cambon, Christine Mac Nab, Sophie Moroge, Claire Rousseau, Vincent Foissaud, Thierry De Greslan, Hervé Granier, Gilles Cellarier, Eric Valade, Philippe Kraemer, Philippe Alla, Audrey Mérens, Emmanuel Sagui, Thierry Carmoi, Christophe Rapp

**Affiliations:** French Military Teaching Hospital Laveran, Marseille, France (H. Savini, P.-Y. Cordier, S. Moroge, P. Kraemer, E. Sagui);; French Military Teaching Hospital Saint Anne, Toulon, France (F. Janvier, J. Bordes, J. Cotte, G. Cellarier, P. Alla);; French Military Teaching Hospital Legouest, Metz, France (L. Karkowski, N. Gagnon);; French Military Teaching Hospital Val de Grâce, Paris, France (M. Billhot, A. Cambon, T. De Greslan, T. Carmoi);; French Military Teaching Hospital Percy, Clamart, France (M. Aletti, J.-M. Cournac, C. Mac Nab, V. Foissaud);; Guinean Military Health Services, Conakry, Guinea (F. Koulibaly);; Frégate Européenne Multi-Mission Aquitaine, Bordeaux Consortium for Regenerative Medicine, Brest, France (N. Maugey);; French Military Teaching Hospital Clermont Tonnerre, Brest, France (C. Rousseau, H. Granier);; Institut de Recherche Biomédicale des Armées, Brétigny sur Orge, France (E. Valade);; French Military Teaching Hospital Bégin, Saint Mandé, France (A. Mérens, C. Rapp)

**Keywords:** Ebola virus disease, Ebola virus, viruses, occupational exposures, treatment center, healthcare workers, Conakry, Guinea

## Abstract

We report 77 cases of occupational exposures for 57 healthcare workers at the Ebola Treatment Center in Conakry, Guinea, during the Ebola virus disease outbreak in 2014−2015. Despite the high incidence of 3.5 occupational exposures/healthcare worker/year, only 18% of workers were at high risk for transmission, and no infections occurred.

Occupational infections during the West Africa Ebola virus disease (EVD) outbreak in 2014–2015 were a major concern because this outbreak caused 109 deaths among the healthcare workers in Guinea (*1*). There was also international concern when secondary cases occurred in Spain and the United States (*2*,*3*).

The Healthcare Workers Treatment Center in Conakry, Guinea, sought to diagnose and treat healthcare workers with suspected or proven EVD by offering extensive medical care (e.g., blood or plasma transfusions, central venous catheterization, biologic monitoring). This center had 5 persons with suspected EVD and 9 persons with confirmed EVD.

The first objective of this study was to describe the occupational exposures occurring in the Healthcare Workers Treatment Center. The second objective was to analyze factors associated with the frequency of high-risk exposures.

## The Study

A total of 66 volunteers from the French Armed Forces Medical Service worked in the high-risk zone for EVD. These volunteers wore personal protective equipment (PPE): coveralls with hoods, large goggles, waterproof respirator masks that filter >94% of airborne particles, waterproof overshoes, a double pair of nitrile gloves, and a third pair of latex gloves. They followed preliminary biosafety training on basic rules, prevention of percutaneous injuries, and management of incidents in exposed or infected areas (e.g., skin exposure, body fluid projection, fainting). Because removal of PPE was considered the highest risk for virus transmission (*4*), we opted for PPE removal by protected persons trained to undress persons without spraying the high-risk zone with bleach.

We conducted a descriptive prospective study during January 23–May 8, 2015, of all occupational exposures in the high-risk zone and reported by a healthcare worker. Occupational exposure was defined as any malfunction of PPE or any noncompliance of biosafety protocols in the high-risk zone. Incidental and demographic data, risk evaluation, and interventions were obtained by using a standardized questionnaire for all reported exposures.

When an exposure occurred, the exposed healthcare worker had to report the exposure to the physician at the Healthcare Workers Treatment Center. This physician used a detailed questionnaire to obtain information on conditions of exposure and evaluated the risk for transmission as low or high, as per French recommendations (Table 1) (*5*). On the basis of results of this evaluation, clinical monitoring or postexposure prophylaxis (PEP) with favipiravir was prescribed. Correlates of risk exposure were examined by using the χ^2^ test for categorical variables and the Mann-Whitney test for continuous variables.

**Table 1:** Risk levels of transmission factors for Ebola virus disease for healthcare workers, Conakry, Guinea*

Exposure	Risk level	
	EVD with diarrhea, vomiting, and hemorrhaging	EVD without diarrhea, vomiting, and hemorrhaging
Contact (>1 m) with patients not projecting biological fluids	Low	Low
Close contact (<1 m) with patients not projecting biological fluids	Low	High
Direct contact with biological fluids	High	High
Cumulative incidents during removal of PPE	Low	High
Transcutaneous or mucosal exposure to infected biological fluids	Maximum	Maximum

*Adapted from recommendations of the French High Council on Public Health (*5*). EVD, Ebola virus disease; PPE, personal protective equipment.

A total of 22 healthcare workers from Guinea with confirmed EVD were treated in the Healthcare Workers Treatment Center during the study. These workers represented 85% of infected healthcare workers from Guinea during the same period. Six of these workers died (mortality rate 27%). None of them worked in an Ebola Treatment Center but all were infected in their community or in other public/private healthcare facilities when not using PPE. During January 23–May 8, 2015, healthcare workers from France at the Healthcare Workers Treatment Center had 3,081 encounters with the high-risk zone for EVD. A total of 77 cases of occupational exposures in the high-risk zone were reported by 57 healthcare workers (30 nurses) from France, which represented an incidence of 2.5% (3.5 occupational exposures/worker/y) (Table 2). Most (62, 80.6%) workers had a low risk for virus transmission.

**Table 2:** Characteristics of 77 occupational exposures for healthcare workers at the Ebola Treatment Center, Conakry, Guinea, January–May, 2015*

Characteristic	Total, n = 77	Low risk, n = 62	High risk, n = 15
Exposure			
Healthy skin >1 m from patient†	52 (67.5)	52 (83.9)	0
Healthy skin <1 m from patient†	11 (14.3)	0	11 (73.3)
Mucous membrane >1 m from patient	1 (1.3)	1 (1.6)	0
Undressing patient	6 (7.8)	6 (9.7)	0
Fluid projection on healthy skin†	2 (2.6)	0	2 (13.3)
Fluid projection on mucous membrane	1 (1.3)	0	1 (6.7)
Percutaneous exposure	0	0	0
Other	4 (5.2)	3 (4.8)	1 (6.7)
Exposed worker activity‡			
Fluid management	26 (33.8)	21 (33.9)	5 (33.3)
Patient care or clinical examination	35 (45.5)	28 (45.2)	7 (46.7)
Blood sampling†	13 (16.9)	7 (11.3)	6 (40.0)
Supervision	6 (7.8)	4 (6.5)	2 (13.3)
Undressing patient	9 (11.7)	9 (14.5)	0
Other	5 (6.5)	4 (6.5)	1 (6.7)
Mean activity duration, min	53.5	53.9	52
Exposure time interval			
6:00 am–10:00 am	30 (39.0)	24 (38.7)	6 (40.0)
10:01 am–4:00 pm	16 (20.8)	11 (17.7)	5 (33.3)
4:01 pm–8:00 pm	21 (27.3)	18 (29.0)	3 (20.0)
8:01 pm–5:59 am	9 (11.7)	8 (12.9)	1 (6.7)
No data	1 (1.3)	1 (1.6)	0
Time of exposure			
First month	56 (72.7)	45 (72.6)	11 (73.3)
Last month	21 (27.3)	17 (27.4)	4 (26.7)

*Values are no. (%) unless otherwise indicated. †Associated with high risk of virus transmission (p<0.05). ‡>1 activity was possible.

The most frequent type of exposure incident (n = 63) was exposure of healthy skin on the face because goggles or respirator masks did not stay correctly in place during patient care. Only 4 healthcare workers reported problems during removal of PPE. Only 14 high-risk occupational exposures were reported; 11 were exposures of healthy skin <1 m from a patient projecting biologic fluid, 2 were projections of biologic fluids to healthy skin, and 1 was fluid projection to mucous membranes. This final incident occurred during discharge of a cured patient who had an undetectable viral load. Percutaneous exposure did not occur during the study period.

Age, sex, carrying glasses, activity, experience with an activity, duration of the activity in the high-risk zone, exposure time, and time of the study were not associated with a higher frequency of high-risk exposure. The only factor associated with high-risk exposure was obtaining a blood sample (p = 0.016). Most (72.7%) occupational exposures occurred during the first month of the study. For all exposures, skin disinfection with 0.05% sodium hypochlorite and monitoring of body temperature were initiated. PEP with favipiravir was not used, and no patients were evacuated to France. EVD did not develop in healthcare workers at the Healthcare Workers Treatment Center during the study or after they returned from Guinea to France.

## Conclusions

To our knowledge, there are few data regarding occupational exposures in a medical facility caring for EVD patients. Limited data are available for potential occupational exposures in an Ebola treatment center (*6*). Rare cases of EVD in healthcare workers have been reported from Africa (*7**−**9*) or other areas (*2*,*3*,*9*). However, all healthcare workers from Guinea who we treated were infected in their communities or when providing care in other healthcare facilities (*7**,**8*). In the Healthcare Workers Treatment Center, we observed a high incidence of 3.5 occupational exposures/healthcare worker/year, which was much higher than the incidence of 0.077 occupational exposures/nurse/year typically observed in hospitals in France (*10*). This high incidence was responsible of excessive concern by some of the healthcare workers from France. However, this concern should be balanced by the low risk for Ebola virus transmission for each exposure.

Classification of transmission risk was difficult. The French recommendations (*5*) were established for exposures in hospitals in France and were not adapted for poorly equipped hospitals (e.g., the Healthcare Workers Treatment Center was composed of tents and direct contact with infected walls was frequent because of lack of space and displacement of googles or masks). Data show that infection with Ebola virus from environment is possible (*11*). More than 80% of occupational exposures were at low risk for virus transmission and did not justify prescription of antiviral treatment, such as favipiravir, which has been used to prevent EVD infection despite lack of data concerning its efficiency (*12*–*14*). A large part of skin exposure should be avoided by improving PPE and limiting activities could displace goggles or masks.

We observed various circumstances that could affect exposure to Ebola virus. In contrast to what we expected (*4*), exposure incidents during removal of PPE were rare, probably because healthcare workers are extensively trained for this activity. Thus, an increase in infections was not observed. No demographic, professional, or incidental factors were associated with a higher frequency of risk exposure. Obtaining a blood sample was a high-risk activity because this can be a stressful procedure and because of constraints associated with PPE, such as an increased core body temperature (*15*).

Technical training for healthcare workers dealing with EVD patients should be increased. A large number of occupational exposures occurred in the first month of the study, which showed that more technical experience could decrease the risk for infection. Despite the high incidence of occupational exposures, no infections occurred during or after the study, which showed that countermeasures we implemented were efficient in preventing virus transmission. Nosocomial transmission of Ebola virus can be avoided by appropriate materials, reliable biosafety protocols, and training. These suggestions could explain why only a few cases of transmission at the Ebola Treatment Center were observed. However, improvements in PPE components, training of healthcare workers, and PEP strategy are required to face future outbreaks of virus diseases.
